# The Variability of the 16S rRNA Gene in Bacterial Genomes and Its Consequences for Bacterial Community Analyses

**DOI:** 10.1371/journal.pone.0057923

**Published:** 2013-02-27

**Authors:** Tomáš Větrovský, Petr Baldrian

**Affiliations:** Laboratory of Environmental Microbiology, Institute of Microbiology of the Academy of Sciences of the Czech Republic, Praha, Czech Republic; University of Waterloo, Canada

## Abstract

16S ribosomal RNA currently represents the most important target of study in bacterial ecology. Its use for the description of bacterial diversity is, however, limited by the presence of variable copy numbers in bacterial genomes and sequence variation within closely related taxa or within a genome. Here we use the information from sequenced bacterial genomes to explore the variability of 16S rRNA sequences and copy numbers at various taxonomic levels and apply it to estimate bacterial genome and DNA abundances. In total, 7,081 16S rRNA sequences were *in silico* extracted from 1,690 available bacterial genomes (1–15 per genome). While there are several phyla containing low 16S rRNA copy numbers, in certain taxa, e.g., the Firmicutes and Gammaproteobacteria, the variation is large. Genome sizes are more conserved at all tested taxonomic levels than 16S rRNA copy numbers. Only a minority of bacterial genomes harbors identical 16S rRNA gene copies, and sequence diversity increases with increasing copy numbers. While certain taxa harbor dissimilar 16S rRNA genes, others contain sequences common to multiple species. Sequence identity clusters (often termed operational taxonomic units) thus provide an imperfect representation of bacterial taxa of a certain phylogenetic rank. We have demonstrated that the information on 16S rRNA copy numbers and genome sizes of genome-sequenced bacteria may be used as an estimate for the closest related taxon in an environmental dataset to calculate alternative estimates of the relative abundance of individual bacterial taxa in environmental samples. Using an example from forest soil, this procedure would increase the abundance estimates of Acidobacteria and decrease these of Firmicutes. Using the currently available information, alternative estimates of bacterial community composition may be obtained in this way if the variation of 16S rRNA copy numbers among bacteria is considered.

## Introduction

rRNA sequences and especially the 16S rRNA represent the most important current targets of study in bacterial evolution and ecology, including the determination of phylogenetic relationships among taxa, the exploration of bacterial diversity in the environment and the quantification of the relative abundance of taxa of various ranks [1]. The 16S rRNA is suitable for this purpose for several reasons. The gene is universally distributed, allowing the analysis of phylogenetic relationships among distant taxa. As a functionally indispensable part of the core gene set, the 16S rRNA gene is expected to be only weakly affected by horizontal gene transfer [2], which further supports its use for phylogenetic studies. Despite the above, 16S rRNA is still subject to variation, especially in certain variable regions. While the presence of variable regions allows sufficient diversification to provide a tool for classification, the presence of conserved regions enabled the design of suitable PCR primers or hybridization probes for various taxa at different taxonomic levels ranging from individual strains to whole phyla [3].

Despite the wide use of 16S rRNA, there are several aspects that limit the interpretation of 16S rRNA-derived results. The most important is the fact that its copy numbers per genome vary from 1 up to 15 or more copies [4]. Copy numbers seem to be taxon-specific to some extent, but variation among strains of the same species has also been recorded [5]. The numbers of rRNA copies have been put into context with the life strategy of bacteria because the rRNA copy number of some taxa are correlated with their ability to respond to favorable growth conditions. Taxa with low copy numbers have been assumed to be more oligotrophic [6], [7].

It is assumed that copies of rRNA genes within an organism are subject to homogenization through gene conversion [8]. Nevertheless, 16S sequences from the same species or even the same genome are often different. Consequently, the amount of 16S rRNA variants was estimated to be 2.5-fold greater than the number of bacterial species [5], and highly dissimilar 16S rRNA sequences were observed in some bacterial taxa [9], [10]. Bacterial species with sequences that differ by >1% are quite common [11]. An even greater variability of 16S rRNA sequences was detected in thermophilic bacteria. In this particular case, the higher incidence of horizontal gene transfer was proposed to be the potential cause [5].

Because the current analysis of bacterial communities most often relies on the construction of similarity clusters (or operational taxonomic units, OTUs) of 16S rRNA gene PCR amplicons, both the multiplicity and variability represent problems for the assessment of bacterial diversity and community structure, i.e., the relative abundance of individual taxa. While the former skews the abundance estimates of individual taxa, the latter affects diversity estimates, and it was recognized early that the lack of information on 16S rRNA copy numbers and genome sizes make the relative abundance estimates in complex bacterial populations unreliable [12]. These problems are not restricted to bacteria because the linking of cell abundance and PCR amplicon abundance is also limited by the multicopy nature and intragenomic variability of the most common molecular marker of fungi, the ITS region of rRNA [13], [14]. In contrast to the PCR amplification-based analyses, shotgun metagenomic data are not immediately affected by the 16S rRNA copy numbers; however, their use for the estimation of bacterial community composition is limited by the variation in genome sizes.

Until recently, it was difficult to draw conclusions about the extent of the biases caused by the use of 16S rRNA analyses; this was primarily due to the limited amount of sequenced bacterial genomes and the absence of these data from certain common phyla [5]. However, recent advances in bacterial genomics allow to analyze the existing dataset in order to answer important questions about the potential of 16S rRNA in bacterial community ecology: Are related taxa more similar in genome sizes/16S rRNA copy numbers? If there are more copies per genome, how similar are they? Are they so distant that it affects diversity estimates using OTU construction? How does this affect the analyses of bacterial community composition using 16S rRNA amplicon analysis? The aim of this paper was to answer the above questions. In addition, we propose the use of currently available genome data for the improvement of the estimates of the relative abundance of bacterial taxa and verify this approach *in silico*.

## Materials and Methods

### *In silico* analysis of sequenced bacterial genomes

For the *in silico* analysis, 1,690 publicly available complete genomes (i.e., those designated as “Complete” with known gene count and genome size) of identified bacterial species were downloaded from GenBank (http://www.ncbi.nlm.nih.gov/genbank/) in February 2012 (Dataset S1). The 16S rRNA sequences were identified by locating the 16S rRNA-targeting reverse and forward primers eub530F and eub1100aR targeting the V4–V6 regions [15], [16] within a distance of 1000 bases on both the plus and minus strands. Regions 900 bases upstream or downstream from this region were retrieved for further processing. In addition, the consensus sequence of the rDNA between the above primers (obtained by the alignment of full-length bacterial 16S rRNA sequences selected from GenBank to represent all phyla with genome sequences available) was searched in the genomes by local BLAST. In the rare cases where the 16S rRNA copy numbers per genome obtained using the latter approach were higher (<0.5% of genomes), the regions around the blast hit were also retrieved. All sequences obtained were aligned with the GenBank 16S rRNA dataset using the RDP pyrosequencing aligner (http://pyro.cme.msu.edu/spring/align.spr) with default settings, and incomplete sequences were manually removed. In total, 7,081 complete 16S rRNA sequences were retrieved. The FASTA file containing these sequences is available as Dataset S2 (unaligned) and Dataset S3 (aligned).

GenBank taxonomy was used to group bacterial species into higher taxonomic ranks-families, classes, and phyla. These assignments were used to characterize the variation in genome sizes and 16S rRNA copy numbers per genomes at various taxonomic levels. At a species level, all genomes of individual strains were analyzed. At higher taxonomic levels, to avoid the effect of variable numbers of sequenced genomes per bacterial species, calculations were performed with mean values for all species belonging to the taxon.

Pairwise similarities of whole 16S rRNA sequences were calculated with a gap opening considered to be equal to mismatch and the gap extension disregarded in BioNumerics 6.5 (Applied Maths, Belgium). Mean pairwise similarities were calculated among all 16S rRNA sequences within each genome, among all pairs of 16S rRNA sequences belonging to different genomes within the same species and among all pairs of 16S rRNA sequences belonging to different species within the same bacterial genus. The within-genome similarity matrices were screened for genomes with at least one pair of 16S rRNA sequences with a pairwise similarity lower than 97%. A tree was constructed from these sequences using the neighbor-joining method with the nucleotide substitution type, the Number of differences model and complete deletion of gaps in the software MEGA 5.05 [17].

Operational taxonomic units (OTUs), the groups of DNA sequences that share defined level of similarity, are often constructed to estimate the diversity of the bacterial community at certain taxonomic levels. We performed OTU construction with the entire 16S rRNA sequences from the Dataset S2 to analyze the relationships between the taxonomic identity of bacterial taxa and these mathematical constructs. OTUs were constructed using CD-HIT [18] at sequence similarity levels of 90%–100%. At all similarity levels, the total number of OTUs was calculated, and the number of species and genera within each OTU were calculated. The taxa denoted as *Escherichia* and *Shigella* were treated as belonging to the same genus.

### Assessment of bacterial genome copy numbers and DNA abundances in amplicon pyrosequencing data

The information on the genome size and 16S rRNA copy numbers per genome were used to estimate the abundance of bacterial genomes and DNA using the previously generated 454-pyrosequencing dataset that contained partial 16S rRNA sequences of the DNA and cDNA from forest topsoil. The sequences originated from a previous study [15] and represented sequences derived from the litter horizon DNA, litter horizon cDNA, soil DNA and soil cDNA (four replicates each). The amplicons were generated using the primers eub530F/eub1100aR and sequenced from the eub530F primer; in total, 204,826 sequences with a length above 300 bp were obtained [15]. Pyrosequencing noise reduction was performed using a Denoiser 0.851 [19], and chimeric sequences were detected using UCHIME [20] and deleted. Sequences were truncated to 300 bases, clustered at a 97% similarity using CD-HIT [18], and consensus sequences were constructed for OTUs with >2 sequences (or representative sequences of OTUs with <3 sequences). For each OTU consensus sequence, the best database hit (this with the lowest E value) was obtained using BLASTn against GenBank [21], while the best genome hit was obtained using the BLASTn against the 16S rRNA dataset derived from bacterial genomes (i.e., Dataset S2). The quality of hits was compared between the best database hit and the best genome hit for each sequence considering the percentage of similarity for hits to >90% of the query sequence length. Phylum assignments were also compared for the two best hits.

The best genome hits were used to calculate the relative abundance of 16S rRNA sequences among bacterial phyla in the samples. For the calculation of the relative abundance of bacterial genomes by phyla, the abundance of sequences belonging to each OTU was divided by the count of 16S rRNA sequences per genome of the best genome hit. To obtain the relative abundance of bacterial DNA by phyla, these values were further multiplied by the sizes of the genomes of the best genome hit.

To analyze the efficiency of the above approach for approximating the genome counts and sizes in the environmental samples, the Dataset S2 was processed as follows: a subset was created that contained one genome from each bacterial species. From this subset containing 909 genomes, 100 genomes were randomly selected. From these genomes, 300-bp sequences, starting with the eub530F primer, were generated, and these sequences were searched in the set of the remaining 809 genomes. For each of the 100 selected genomes, the actual genome size and 16S rRNA copy number were compared to the genome size estimates and 16S rRNA copy number estimates obtained (1) as the values of the genomes with the closest hit; (2) as the average values of the bacterial phylum to which the genome belonged; and (3) as a mean of all genomes (i.e., considering equal size and 16S rRNA copy numbers for all genomes). The sets of these estimates were compared.

One-way ANOVA with the Fisher LSD post-hoc test was used to analyze the significant differences among datasets (e.g., 16S rRNA gene copy numbers or the abundance of fungal genomes in soil among phylogenetic groups). Differences of P<0.05 were regarded as statistically significant.

## Results

### *In silico* analysis of sequenced bacterial genomes

**Figure 1:**
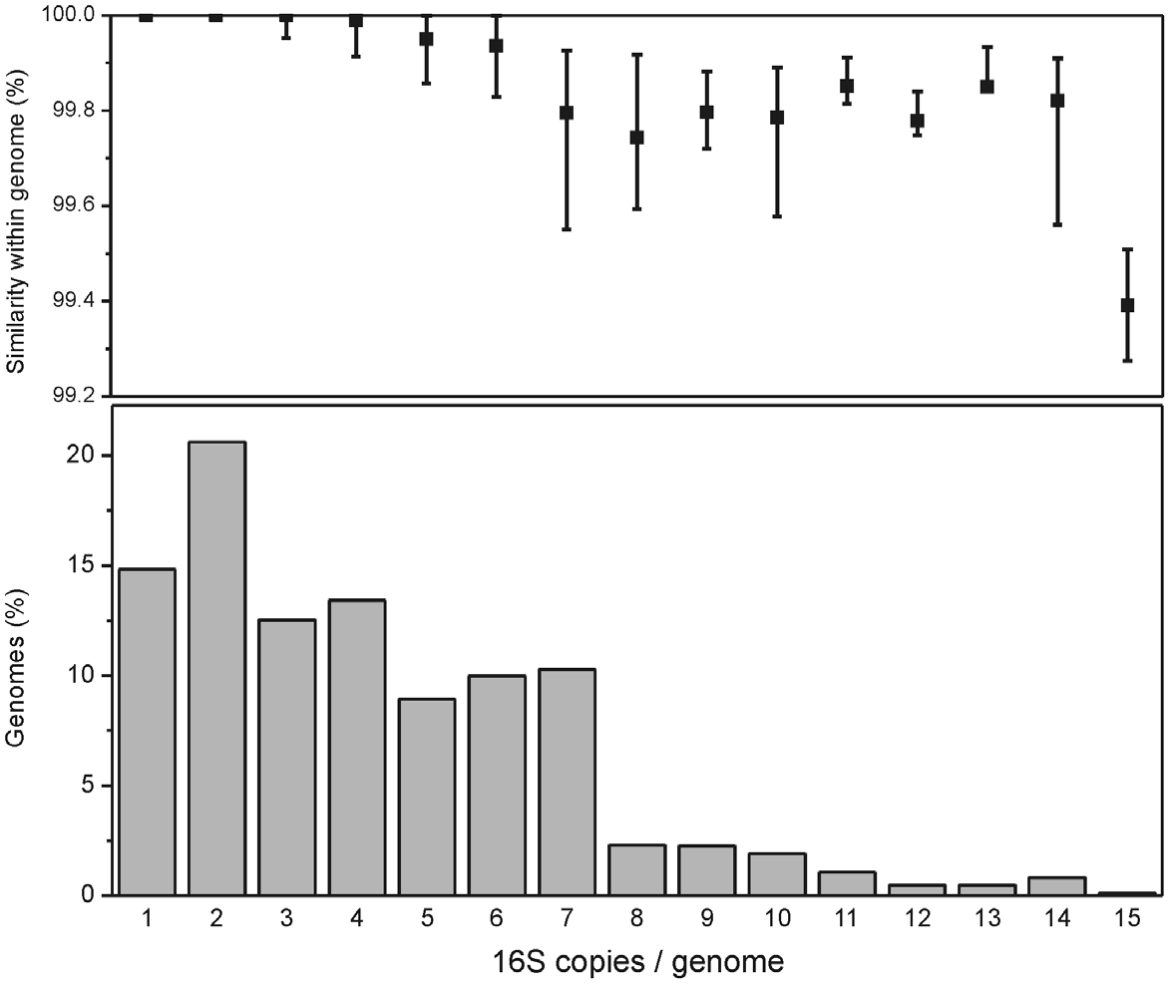
16S rRNA within-genome similarity and copy numbers in bacterial genomes. Upper panel: the similarity of genomes with various copy numbers: the values indicated represent the first, the second and the third quartile. Lower panel: distribution of 16S rRNA copy numbers per genome in 1,690 sequenced bacterial genomes.

Of a total of 1,690 available bacterial genomes that belonged to 909 identified bacterial species and 454 genera, most available genomes belonged to the Proteobacteria (especially Gammaproteobacteria, Alphaproteobacteria and Betaproteobacteria), Firmicutes, and Actinobacteria with >100 genomes per phylum; the phyla Caldiserica, Elusimicrobia, Gemmatimonadetes, Ignavibacteria, and Thermodesulfobacteria were represented by only one genome each (Table 1). A total of 7,081 16S rRNA copies were identified in, with an average of 4.2 copies per genome. Fifteen percent of genomes contained a single 16S rRNA copy, while 21% contained two copies, and 3–7 16S rRNA copies per genome were frequently found. Copy numbers above 7 were relatively rare, and the maximum value was 15 (Figure 1).

**Table 1:** Overview of bacterial genomes and their properties at the phylum level (class level for the Proteobacteria).

Phylum	Genera	Species	Genomes	16S rRNA/genome	Genome size (Mb)
Acidobacteria	3	4	4	1.0±0.0	5.24±0.88
Actinobacteria	66	117	201	3.1±1.7	5.03±2.53
Aquificae	6	6	6	2.0±0.6	1.63±0.11
Bacteroidetes	37	52	59	3.5±1.5	4.51±1.83
Caldiserica	1	1	1	1	1.56
Chlamydiae	5	11	72	1.4±0.5	1.54±0.72
Chlorobi	5	10	11	1.7±0.7	2.59±0.41
Chloroflexi	9	10	10	2.2±1.2	4.12±1.73
Cyanobacteria	10	11	23	2.3±1.2	5.66±2.45
Deferribacteres	4	4	4	2.0±0.0	2.63±0.42
Deinococcus-Thermus	6	13	16	2.7±1.0	3.12±0.67
Dictyoglomi	1	2	2	2.0±0.0	1.91±0.07
Elusimicrobia	1	1	1	1	1.64
Fibrobacteres	1	1	2	3	3.84
Firmicutes	69	186	395	5.8±2.8	3.09±1.18
Fusobacteria	5	5	5	5.0±0.7	2.79±1.09
Gemmatimonadetes	1	1	1	1	4.64
Ignavibacteria	1	1	1	1	3.66
Nitrospirae	2	2	2	2.0±1.4	2.28±0.39
Planctomycetes	5	6	6	1.7±0.8	5.71±1.07
Proteobacteria					
Alphaproteobacteria	58	112	179	2.2±1.3	3.58±2.01
Betaproteobacteria	39	66	101	3.3±1.6	5.09±2.18
Deltaproteobacteria	25	37	43	2.7±1.4	4.95±2.56
Epsilonproteobacteria	9	23	70	3.0±1.1	1.97±0.41
Gammaproteobacteria	78	157	379	5.8±2.8	4.23±1.25
Spirochaetes	5	20	31	2.4±1.0	3.16±0.95
Synergistetes	4	4	4	2.5±1.0	1.88±0.15
Tenericutes	4	25	43	1.6±0.5	0.93±0.20
Thermodesulfobacteria	1	1	1	2	2.32
Thermotogae	6	13	13	1.8±1.0	2.02±0.17
Verrucomicrobia	4	4	4	1.8±1.0	3.66±1.65

**Figure 2:**
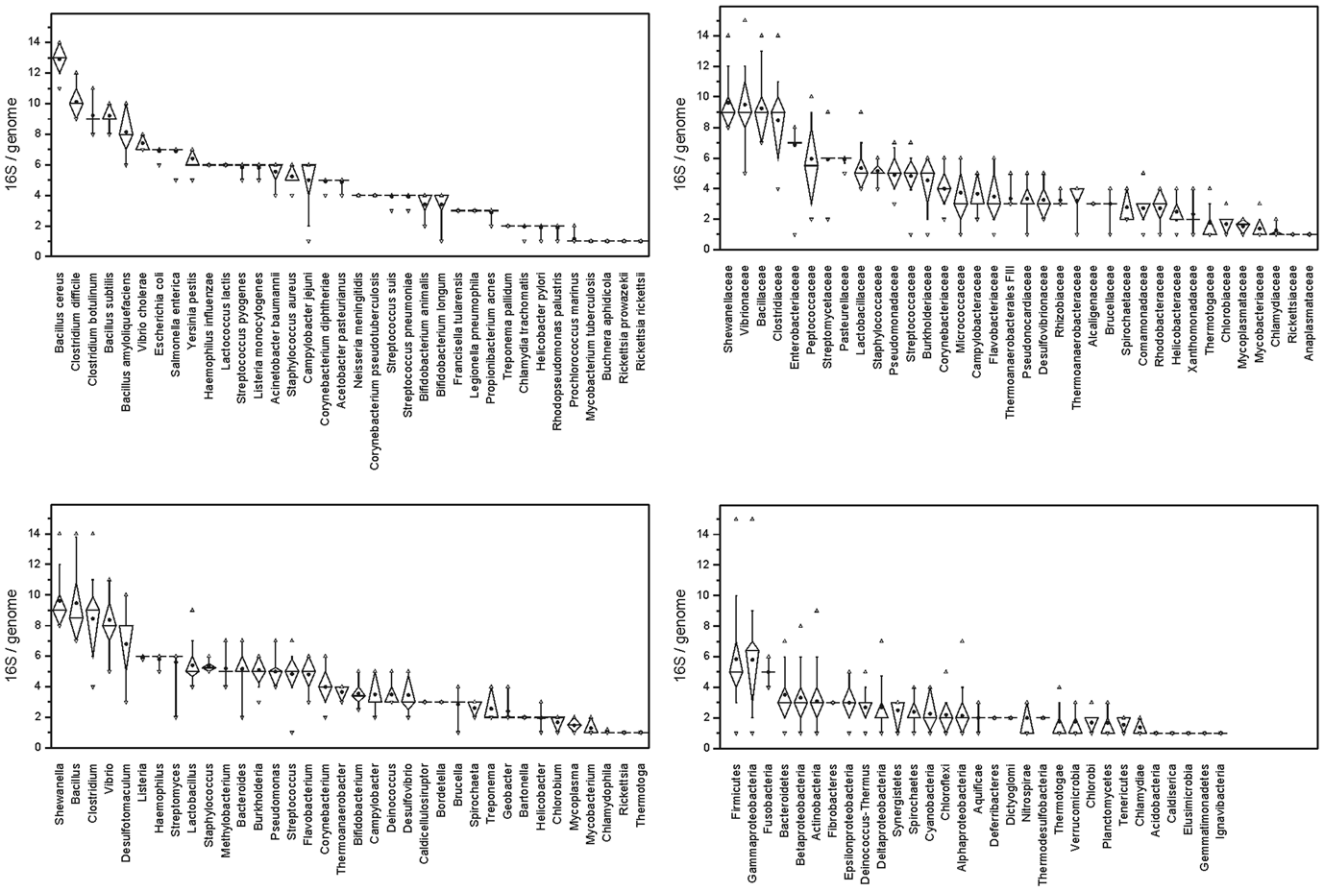
16S rRNA copy numbers in bacterial genomes. 16S rRNA copy numbers in bacterial phyla (classes), selected families (n>6), genera (n>3), and species (n>6). Open triangles indicate minima and maxima, whiskers the 10th and 90th quantile and boxes the 25th and 75th quantile. Median is indicated as a horizontal line and mean as a dot.

The 16S rRNA copy numbers per genome were taxon-specific at several taxonomical levels (Figure 2, Figure S1). They were especially high in the Firmicutes, Gammaproteobacteria and Fusobacteria, with averages per genome of above five. However, in the former two groups, copy numbers varied widely between 1 and 15. One 16S rRNA copy per genome was present in Caldiserica, Elusimicrobia, Gemmatimonadetes, Ignavibacteria and Acidobacteria, but only the latter group was represented by more than one genome sequence. Most bacterial phyla showed wide variation in 16S rRNA copy numbers, with most containing at least one representative with a single 16S rRNA copy. At lower taxonomic levels, the variation of 16S rRNA copy numbers was less pronounced, and differences at the level of families, genera and species were often statistically significant (data not shown). Among bacterial families with available genomes from more than eight different species, several exhibited high 16S rRNA copy numbers: all members of the Shewanellaceae, Vibrionaceae, Bacillaceae, and Pasteurellaceae contained more than five copies. The 16S rRNA copy numbers were even more conserved at the level of bacterial genera and species. For the top 36 bacterial taxa (families with >6 species, genera with >3 species, or species with >6 genomes), the coefficients of variation (standard deviations divided by means) of the 16S rRNA copy numbers were 29% on the family level, 22% on the genus level and 9.2% on the species level. However, some species, e.g., *Bacillus amyloliquefaciens*, *Campylobacter jejuni* or *Bifidobacterium longum*, showed high interspecific variability in the 16S rRNA copy numbers (Figure 2).

**Figure 3:**
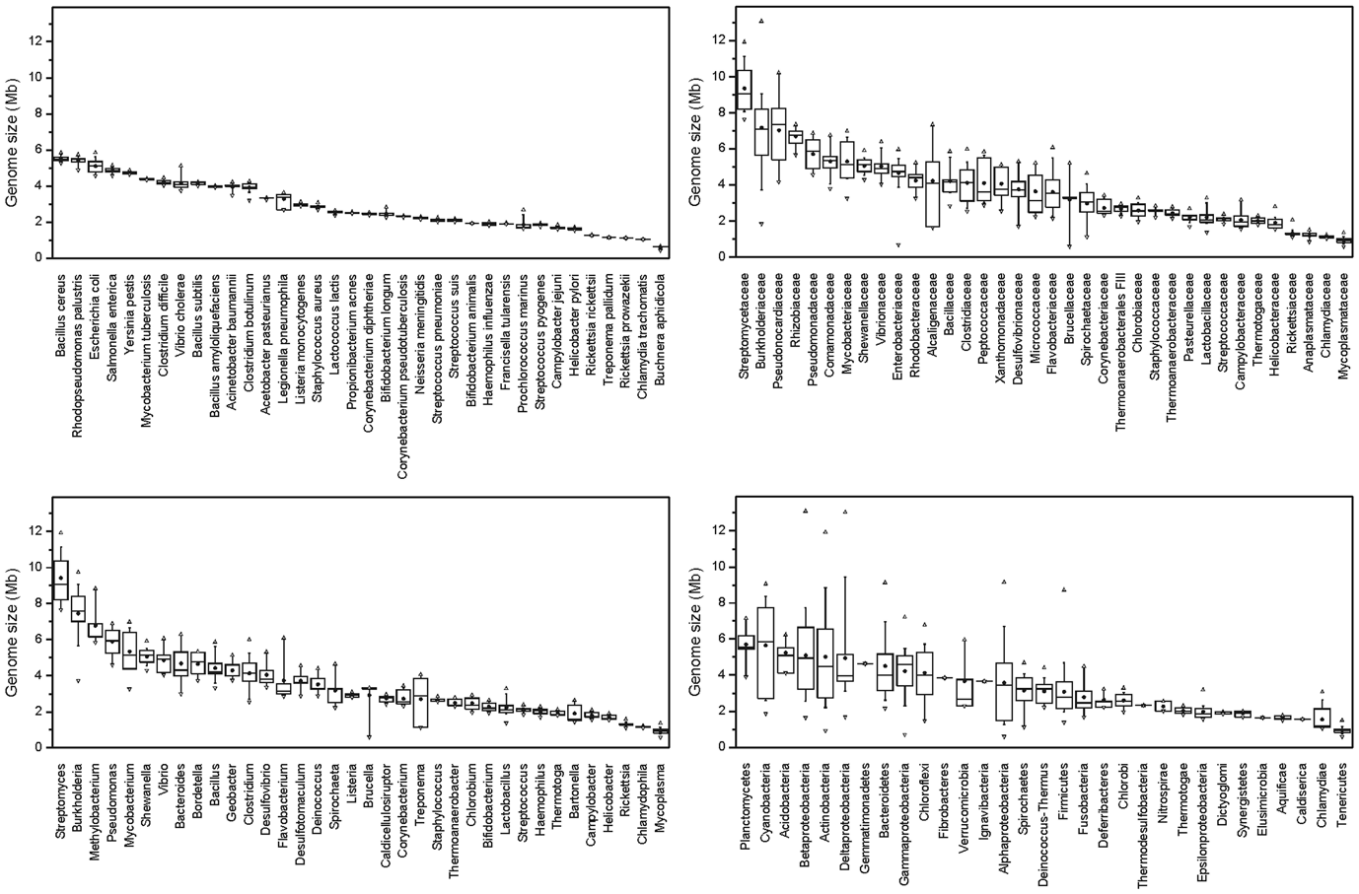
Sizes of bacterial genomes. Sizes of genomes in bacterial phyla (classes), selected families (n>6), genera (n>3), and species (n>6). Open triangles indicate minima and maxima, whiskers the 10th and 90th quantile and boxes the 25th and 75th quantile. Median is indicated as a horizontal line and mean as a dot.

Furthermore, the sizes of bacterial genomes were taxon-specific at all studied taxonomical levels (Figure 3). Several bacterial phyla (or classes of Proteobacteria) harbored small genomes with low size variation: the smallest genomes were found in Tenericutes (0.93±0.20) and Chlamydiae (1.54±0.72). However, several groups widely differed in genome sizes, e.g., the Actinobacteria, with sizes from 1 to 12 Mb, as well as the Beta- and Deltaproteobacteria (2–13 Mb) and the Cyanobacteria (2–9 Mb). At lower taxonomic levels, the size of genomes was less variable then the 16S rRNA copy numbers: the coefficients of variation were 19% on the family level and 16% on the genus level. Within individual bacterial species, genome size was highly conserved, with a variation lower than 3.8% (Figure 3).

**Figure 4:**
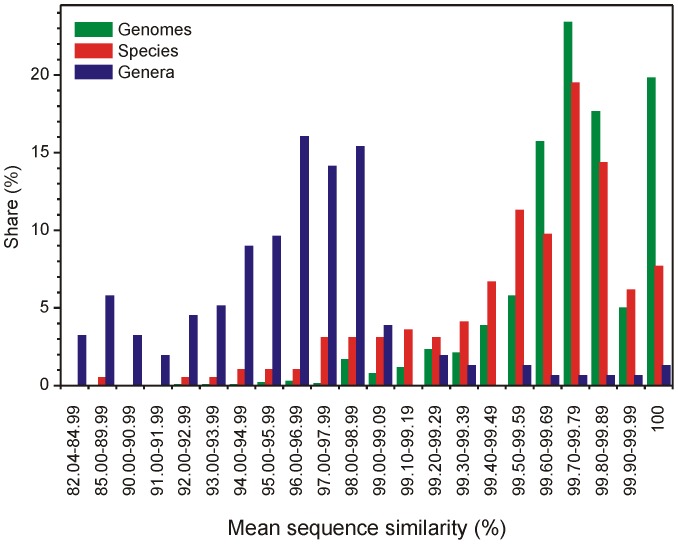
Mean pairwise sequence similarity of 16S rRNA sequences. Similarity within bacterial genomes, among genomes belonging to the same species and among species belonging to the same genus.

16S rRNA is currently the most common target of analysis of the diversity of bacterial communities. Because the most common approach is based on similarity clustering, we explored the variability of the 16S rRNA at various taxonomic levels. At a genome level, 19.8% of all genomes with more than one 16S rRNA copy harbor 2–5 identical 16S rRNA copies, and the average 16S rRNA similarity within a genome is 99.70±0.46%, with 97.6% of genomes showing average 16S rRNA similarity above 99%. The similarity of genomes decreases with 16S rRNA copy numbers, and all genomes with six and more copies harbored at least two different variants of 16S rRNA (Figure 1). Phylogenetically, Firmicutes and Gammaproteobacteria showed high 16S rRNA within-genome variability, while Alphaproteobacteria and Chlamydiae showed low variability; the differences were statistically significant. The level of dissimilarity within a genome can be relatively high: fourteen genomes contained at least one pair of 16S rRNA sequences with a similarity below 97% (Figure 4). These diverse genomes were found in the genera *Clostridium*, *Desulfitobacterium*, *Desulfosporinus*, *Desulfotomaculum*, *Photobacterium*, *Salmonella*, *Selenomonas*, *Syntrophomonas*, *Thermoanaerobacter*, *Thermoanaerobacterium*, and *Thermobispora*. While some of the genomes contained groups of similar 16S rRNA sequences (e.g., the *Thermobispora bispora* DSM 43833 and *Clostridium* sp. BNL 1100), others contained several sequences with low pairwise similarity (Figure S2).

On the level of bacterial species, average 16S rRNA similarity was 99.30±1.38%, and 95.4% of all genomes had mean 16S rRNA similarity over 97%. Among genera, the average 16S rRNA similarity of genomes belonging to different species was 95.56±3.68%. In 12.2% and 41.7% of genera, 16S rRNA similarities among species were higher than 99% and 97%, respectively, while 12.2% of genera contain species with mean pairwise 16S rRNA similarity below 90% (Figure 4).

**Figure 5:**
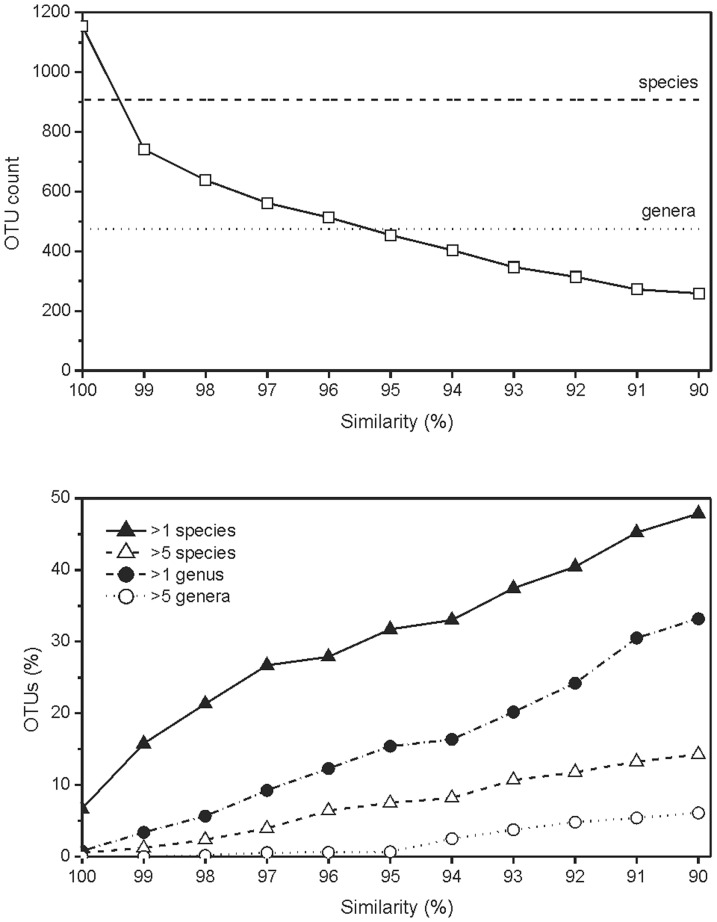
OTU counts and the percentage of OTUs harboring multiple bacterial taxa at various levels of 16S rRNA similarity. Upper panel: OTU counts at various levels of 16S rRNA similarity; the dashed line and the dotted line indicate the number of bacterial species and genera in the Dataset S2. Bottom panel: the percentage of OTUs harboring 16S rRNA sequences belonging to multiple bacterial species or genera.

The fact that the level of 16S rRNA similarity among bacterial species within a genus varied widely poses a question about the reliability of diversity estimates based on the construction of operational taxonomic units by similarity clustering, as well as about the phylogenetic relatedness within the OTU. Ideally, OTUs of certain similarity cutoffs should contain sequences belonging to bacteria of certain, defined taxonomic rank. To test the potential of OTU clustering to meet these criteria, OTUs were constructed at various levels of sequence similarity and analyzed for the number of bacterial species and genera that they contain. Clustering at 99% similarity and 95% similarity gave OTU counts that best corresponded to the species and genus counts, 906 and 471, respectively (Figure 5). However, even at 100% sequence similarity, 6.7% of OTUs contained sequences that belonged to multiple species (up to nine). At a 97% similarity level, 21.3% of OTUs contained sequences of multiple species, and 9.3% contained sequences of multiple genera (Figure 5). At a 95% similarity level, a large majority of OTUs was still represented by sequences belonging to the same genus (84.6%), while the amount of OTUs containing more than two genera was still relatively low (6.8%).

### Assessment of bacterial genome counts and DNA abundances in amplicon pyrosequencing data

The data on the relative abundances of the 16S rRNA sequences in PCR amplicons derived from metagenomic soil DNA were used to estimate the abundance of bacterial genomes (i.e., cells) and DNA. To achieve this, data on 16S rRNA sequence copy numbers of individual OTUs were combined with the predicted 16S rRNA copy numbers and genome sizes, i.e., the values found in the closest known relatives with sequenced genomes. The dataset contained 93,806 sequences that clustered into 15,421 OTUs at a 97% similarity level. When best hits were generated for each OTU, the best hits obtained by comparison with GenBank showed an average 93.2±3.6% similarity with the query, while hits against the genome-derived sequences showed 90.3±3.9% similarity. A different phylum (or class within the Proteobacteria) was assigned to 4.2% of OTUs, most of which has a low similarity to database entries.

**Figure 6:**
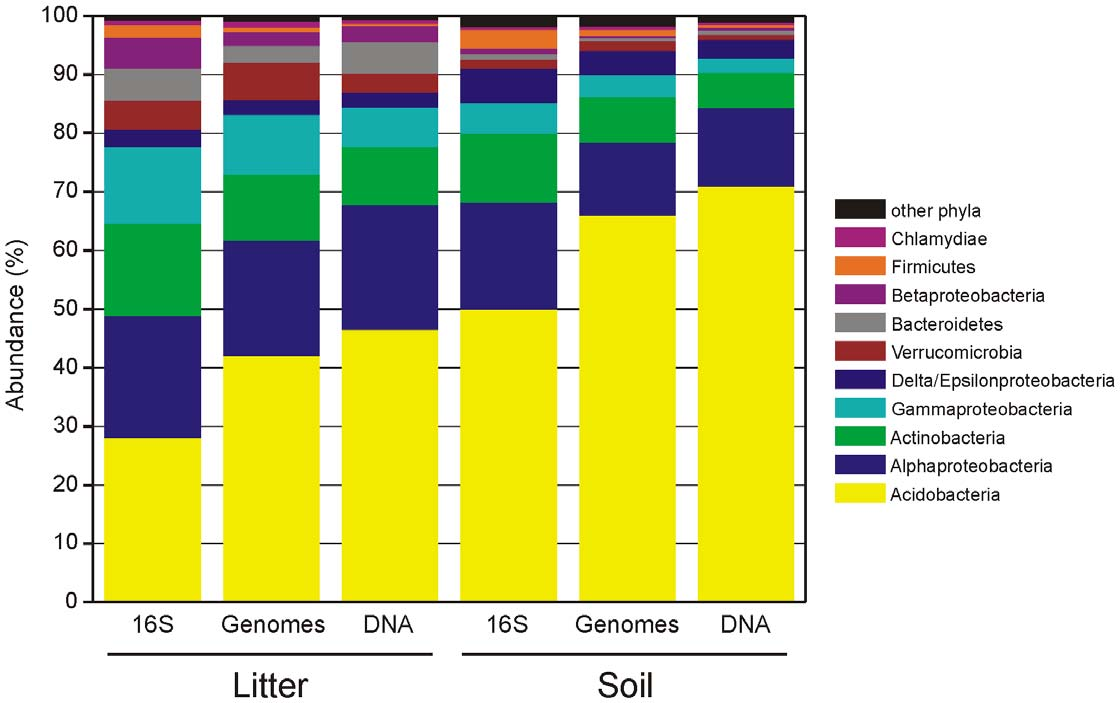
Abundance of bacterial 16S rRNA sequences, genomes and DNA in forest litter and soil. Relative abundance of bacterial 16S rRNA sequences in the amplicon pool from *Picea abies* litter and soil (Baldrian et al., 2012), and estimates of the relative abundance of bacterial genomes and DNA. The estimates were calculated using the values of 16S rRNA copy numbers and genome sizes of the closest hits to each bacterial OTU.

In the original dataset, the 16S rRNA sequences of the Acidobacteria, Alphaproteobacteria and Actinobacteria were the most abundant, representing 28.1%, 20.9% and 15.7%, respectively, of sequences in litter and 50.1%, 18.2% and 11.8%, respectively, of sequences in soil. The estimated genome (cell) abundances showed higher share of the Acidobacteria (42.1% in litter and 66.0% in soil) and a lower share of the Actinobacteria (11.3% and 7.6%). The Acidobacteria were even more represented in the total DNA (46.6% and 71.0%). In addition to Acidobacteria, Chlamydiae, Gemmatimonadetes, and Elusimicrobia also showed substantially higher representation among genomes than among 16S rRNA sequences, while the Bacteroidetes, Betaproteobacteria and Firmicutes were less represented (Figure 6). Although the Firmicutes represented 3.2% of 16S rRNA sequences in soil, they were predicted to compose only 1.1% of genomes. In addition, the relative abundance and ranks of individual OTUs also differed substantially when expressed as 16S rRNA or genome abundances (Table S1).

To explore whether the best hits in the database of sequenced genomes provide reliable estimates of 16S rRNA copy numbers and genome sizes, the best hits for the subset of 100 randomly chosen 16S rRNA sequences were retrieved from the dataset containing 16S rRNA sequences from other bacterial species. The best hits gave significantly better estimates of 16S rRNA copy numbers compared to the means of all genomes (P = 0.0002, better estimate in 75 cases) and also compared to phylum averages (P = 0.0058, 63 cases). This approach also gave better estimates of genome size compared to the grand average (P = 0.0011, 67 cases) or phylum average (P = 0.0443, better estimate in 63 cases). Using best hits, 16S rRNA copy numbers and genome sizes were estimated with average difference of 24% and 25%; the errors were substantially higher when the phylum average was used as the estimate (38% and 32%) or when the grand average was used (48% and 41%). It can therefore be concluded that the use of the best hit in sequenced genomes significantly and substantially improves the estimates of bacterial genome and DNA abundances compared to the abundances in the original 16S rRNA dataset.

## Discussion

Although some 15% of bacterial genomes contain only a single 16S rRNA copy, it seems that most bacterial phyla may contain bacteria with more than one copy and one half of the currently analyzed genomes harbor five or more copies. 16S rRNA copy numbers have been found to show both narrow and wide variation within the tested bacterial genera [22], [23]. Here, we show that the variability in copy numbers remains high even at the level of bacterial families and genera, but despite some exceptions, especially the species with high 16S rRNA copy numbers, they seem to be conserved at the species level. No statistically significant relationship between genome size and 16S rRNA copy numbers were recorded, but, interestingly, the genome size is much better conserved at all levels of phylogenetic classification.

Only a minority of bacterial genomes with multiple 16S rRNA copies carry identical copies of the gene, although the variation is usually minute. Unsurprisingly, the genomes with more 16S rRNA copies tend to carry more diverse variants of the gene. Among the 568 bacterial species analyzed by [11], 16S rRNA sequences with >1% difference were found in 10% of genomes. Here, we show that 2.4% of genomes have 16S rRNA sequences with <99% mean similarity. Genomes with higher dissimilarity are thus relatively rare: although some distant variants may theoretically evolve into pseudogenes, highly dissimilar 16S rRNA sequences from the same genome were demonstrated to still carry a conserved secondary structure [11] and thus seem to be functional and as such kept in the genomes. The existence of highly dissimilar 16S rRNA sequences in certain genomes and the fact that 16S rRNA similarity within such genome can be lower than among certain bacterial genera (as seen for *Desulfitobacterium youngiae*/*D. hafniense* in the Figure S2) may speak in favor of their evolution by horizontal gene transfer.

The diversity of 16S rRNA within a genome is frequently reported as a factor that can potentially increase diversity estimates obtained by OTU construction; however, that the resolution of the 16S rRNA gene is often too low to allow the differentiation of closely related species [24], [25] is often overlooked. Here, we show that this interspecific or intergeneric similarity can also significantly affect OTU construction: in certain genera, member species had exactly the same 16S rRNA sequence (Figure 4). If the standard level of 16S rRNA similarity of 97% is used, a few species, even genomes, can fall to different OTUs due to intragenomic or intraspecific differences. In contrast, species of several genera will not be separated at the same similarity level because in 41.7% of genera, the 16S rRNA differences were lower than 97%. This, however, depends on the part of the 16S rRNA gene used for OTU construction. We should also note that our data were based on bacterial species/genera names that are currently valid. It is unclear how precisely the present definitions of existing bacterial species and genera comply with a biological concept of species definition. This discussion should be opened, and there is also a space for the use of 16S rRNA for this purpose, e.g., for the reclassification of polyphyletic taxa.

Using an example of forest soil bacteria, we show that the recently widely applied 16S rRNA-based abundance estimates provide an imperfect description of bacterial community composition. In general, abundance estimates based on the 16S rRNA sequence counts tend to underestimate the abundance of taxa with low 16S rRNA copy numbers such as the Acidobacteria and to overestimate taxa with high 16S rRNA copy numbers such as Gammaproteobacteria and Firmicutes. This fact should be carefully considered when interpreting the 16S rRNA abundance results. For example, the abundance of 16S rRNA sequences of Acidobacteria in certain organic-rich low-pH soils was found to be over 60% [26]. Based on our results, it is possible that their dominance in genome counts is even greater than this, and they may largely dominate in such environments. We also demonstrate that the currently available genome data can substantially change our estimates of bacterial community composition in environmental samples by providing estimates of genome counts that are ecologically more relevant measures of abundance. Caution must be taken, however, when interpreting estimates such as the counts of bacterial cells. Several bacteria, e.g., the hyperthermophile *Thermus thermophilus* or the vegetative cells of filamentous cyanobacteria, were demonstrated to be polyploidic, with 4–18 genome copies per cell [27], [28]. In some cases, genome counts per cell can be even considerably higher-over 100 in the cyanobacterial akinetes [27] or the large marine species of *Epulopiscium* [29]. Moreover, even if the abundance estimates are improved as demonstrated, they will be still affected by a wide set of potential biases associated with other steps of the experimental procedures including the variation of DNA extraction efficiency among taxa, differences in PCR amplification or random PCR errors (for more information, see e.g. [13]). More research is definitely needed to reduce these sources of uncertainity.

In the past, several single copy housekeeping genes, e.g., those coding for RNA polymerase, ribosomal proteins or amino-acyl synthetases, or the 60 kDa chaperonin have been proposed as potential phylogenetic markers [30]–[34], theoretically avoiding the problems with multiple and variable 16S rRNA copies within bacterial genomes. Indeed, it has been documented that the phylogenetic resolution of *cpn60* or *rpoB* genes can be comparable or even better than that of the 16S rRNA [34]–[35]. Protein-coding single copy genes were also successfully used for the assignment of metagenomic sequences, with a better taxonomic resolution than 16S rRNA fragments [36]–[37]. However, the use of these genes for the analysis of environmental amplicons is limited by the fact that the degeneracy of protein sequences make the design of suitable primers difficult. Although universal primers have been designed for the *cpn60* gene [34], the abundance of 16S rRNA gene sequences in the sequence databases greatly exceeds those of other bacterial genes, which still makes it use preferable by increasing the probability of finding a close hit for taxon identification.

Although the 16S rRNA remains still the target of choice for studies in bacterial ecology, this paper demonstrates that there are limitations in its use for community studies and shows how the current developments in bacterial genomics helps to make bacterial abundance estimates more accurate. We strongly believe that in the very near future, the increasing amount of sequenced bacterial genomes allows even better predictions and our knowledge of bacterial community ecology will significantly increase.

## Supporting Information

Figure S1 **Distribution of 16S rRNA copy numbers in bacterial genomes belonging to selected phyla (classes).** The numbers in parentheses indicate numbers of genomes in the respective groups.
(PDF)

Figure S2 **Neighbor-joining tree of 16S rRNA sequences from bacterial genomes where the pairwise similarity of at least one 16S rRNA pair within a genome was lower than 97%.**
(PDF)

Table S1 Top ten most abundant bacterial OTUs in *Picea abies* forest soil according to the 16S rRNA amplicon sequence copy numbers and estimated genome counts.
(XLS)

Dataset S1 Overview of bacterial genome sequences used in this study.
(XLS)

Dataset S2 Fasta file containing all identified 16S rRNA sequences from bacterial genomes used in this study.
(FAS)

Dataset S3 Fasta file containing aligned 16S rRNA sequences from bacterial genomes used in this study.
(FAS)
