# Supplementary material for: The Variability of the 16S rRNA Gene in Bacterial Genomes and Its Consequences for Bacterial Community Analyses
Source: PLoS One. 2013 Feb 27;8(2):e57923. doi: 10.1371/journal.pone.0057923 (PMC3583900; doi:10.1371/journal.pone.0057923)
Supplement: Figure S1 — Distribution of 16S rRNA copy numbers in bacterial genomes belonging to selected phyla (classes). The numbers in parentheses indicate numbers of genomes in the respective groups. (PDF) [file pone.0057923.s001.pdf]

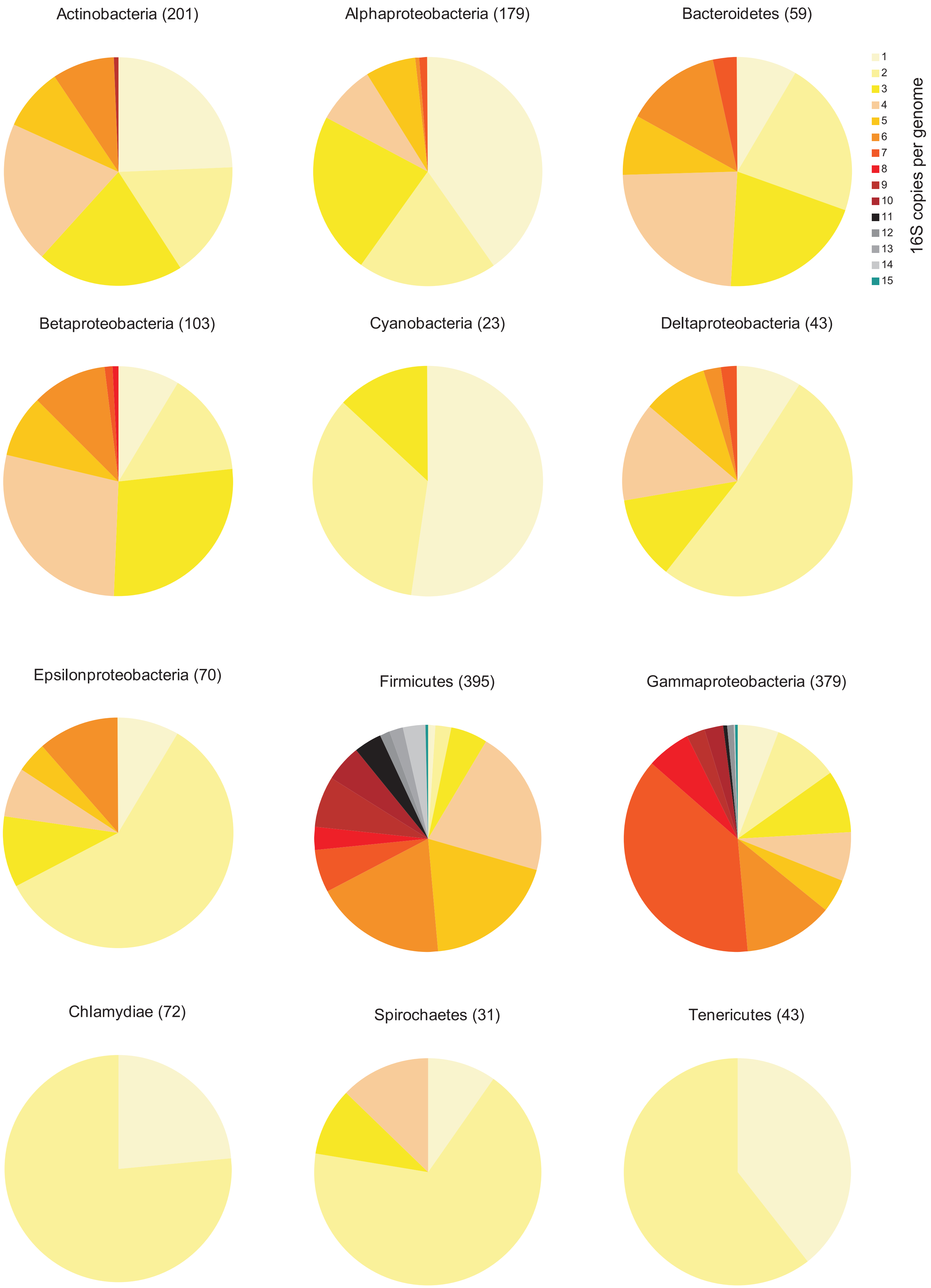
Actinobacteria (201)
Alphaproteobacteria (179)
Bacteroidetes (59)
Betaproteobacteria (103)
Cyanobacteria (23)
Deltaproteobacteria (43)
Epsilonproteobacteria (70)
Firmicutes (395)
Gammaproteobacteria (379)
Chlamydiae (72)
Spirochaetes (31)
Tenericutes (43)
1
2
3
4
5
6
7
8
9
10
11
12
13
14
15
16S copies per genome
