# Supplementary figures and images for: The Variability of the 16S rRNA Gene in Bacterial Genomes and Its Consequences for Bacterial Community Analyses
Source: PLoS One. 2013 Feb 27;8(2):e57923. doi: 10.1371/journal.pone.0057923 (PMC3583900; doi:10.1371/journal.pone.0057923)

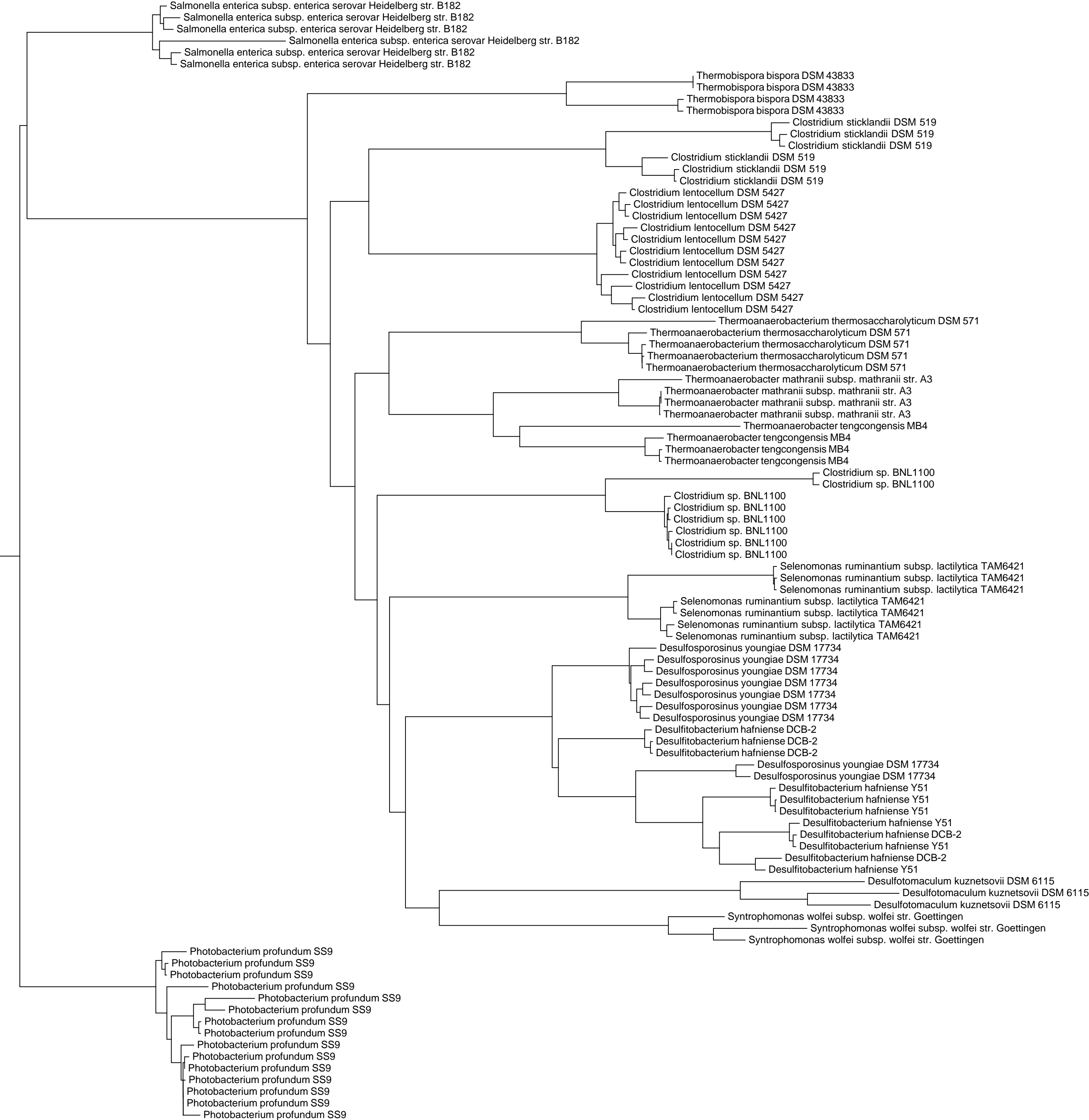

Supplement: Figure S2 — Neighbor-joining tree of 16S rRNA sequences from bacterial genomes where the pairwise similarity of at least one 16S rRNA pair within a genome was lower than 97%. (PDF) [file pone.0057923.s002.pdf]
